# Experimental longitudinal evidence for causal role of social media use and physical activity in COVID-19 burden and mental health

**DOI:** 10.1007/s10389-022-01751-x

**Published:** 2022-09-02

**Authors:** Julia Brailovskaia, Verena J. Swarlik, Georg A. Grethe, Holger Schillack, Jürgen Margraf

**Affiliations:** Mental Health Research and Treatment Center, Department of Clinical Psychology and Psychotherapy, Ruhr-Universität Bochum, Massenbergstr. 9-13, 44787 Bochum, Germany

**Keywords:** Social media use reduction, Physical activity increase, COVID-19 burden, Mental health, Lifestyle, Experimental longitudinal study

## Abstract

**Aim:**

The COVID-19 outbreak has severely impacted people’s mental health. The present experimental study investigated how to reduce this negative effect by a combination of two interventions.

**Subject and methods:**

Participants (*N*_total_ = 642) were users of social media in Germany. For two weeks, the social media group (*N* = 162) reduced its social media use (SMU) by 30 minutes daily, the physical activity group (*N* = 161) increased its physical activity by 30 minutes daily, the combination group (*N* = 159) followed both instructions, and the control group (*N* = 160) did not get specific instructions. Online surveys assessed variables of SMU, physical activity, mental health, COVID-19 burden, and lifestyle at six measurement time points up to six months after the intervention.

**Results:**

In the experimental groups, (addictive) SMU, depression symptoms, and COVID-19 burden decreased, while physical activity, life satisfaction, and subjective happiness increased. All effects were stronger and more stable in the combination group in the longer-term. Smoking behavior decreased in the social media group only.

**Conclusion:**

Thus, the conscious combination of less SMU and more physical activity leads causally to more psychological resilience against negative pandemic impacts and to higher levels of mental health over six months. Prevention programs could improve their effectiveness by integrating the time- and cost-efficient interventions – separately or in combination.

**Supplementary Information:**

The online version contains supplementary material available at 10.1007/s10389-022-01751-x.

## Introduction

“Digitalization” is a main characteristic of the twenty-first century. It represents a transformation of society to an intensive use of digital technologies in various areas of life (Gray and Rumpe 2015). Daily use of social media (SM) such as Facebook, Twitter, Instagram, TikTok, and WhatsApp is a central aspect of this transformation (Parviainen et al. 2017).

The global outbreak of the coronavirus disease 2019 (COVID-19; World Health Organization 2021) resulted in significant changes in people’s daily routine. Lockdowns, curfews, and restrictions of offline social contacts (“social distancing”) sped up the digital transformation in various sectors (Nagel 2020). Social media use (SMU) became one of the main options for social interaction and pastime (Mano 2020). Moreover, some people experience the COVID-19 situation as a heavy psychological burden, which results in enhanced anxiety, uncertainty, and hopelessness (Ornell et al. 2020). Due to lacking alternatives, they often tend to excessive SMU that can reduce negative feelings and provide relief at least temporarily. This can foster one’s perception of SM as a significant source of happiness and satisfaction and thus contribute to further intensive use (Brailovskaia and Margraf 2021).

Following available literature, excessive SMU can negatively influence mental health in the longer-term (Shakya and Christakis 2017). Use of SM as a COVID-19 information source fosters psychological burden due to fake news and conspiracy theories (Zhong et al. 2021). Intensive active exchange with other users can evoke the perception of social support, which fosters the development of an emotional bond to SM (Zhao and Zhou 2021). This bond is closely linked to an obsessive need to stay permanently online, even if the use interferes with obligations and causes interpersonal conflicts (Sun and Zhang 2020). This phenomenon has been termed as addictive SMU and was defined by six typical characteristics (salience, tolerance, mood modification, relapse, withdrawal, conflicts) (Andreassen et al. 2016). Addictive SMU is not a recognized formal psychiatric disorder (e.g., International Classification of Diseases, ICD-11; World Health Organization 2018). However, it is positively linked to enhanced COVID-19 burden (Brailovskaia and Margraf 2021) and low levels of mental health (Sun and Zhang 2020). Notably, recent studies described a decrease of mental health during the pandemic (Bueno-Notivol et al. 2020). Considering the uncertain duration of the COVID-19 situation, the question arises how to protect mental health in times of social distancing and rapid digital transformation. Specifically, which cost- and time-efficient strategies can be implemented in one’s everyday life?

Experimental longitudinal studies that allow true causal conclusions can contribute to the response to this question. The few available experimental research on SMU conducted prior to the pandemic showed that a one-week waiving of SMU significantly improved life satisfaction (Tromholt 2016). A three-week reduction of time spent on SMU up to 30 minutes a day – less challenging than abstinence – decreased depression and anxiety symptoms (Hunt et al. 2018); a two-week reduction of SMU for 20 minutes a day enhanced life satisfaction and decreased addictive use tendencies, depression symptoms, and smoking behavior (Brailovskaia et al. 2020b). Thus, a starting point might be a conscious and controlled change of SMU time. However, would this be enough considering the high involvement of online activity in daily life, especially during the pandemic?

Brailovskaia et al. (2020b) described a spontaneous increase of the frequency of physical activity (e.g., jogging, cycling) among their participants. The authors hypothesized that due to the reduced SMU time, the participants chose physical activity as an alternative source for mood modification. The World Health Organization (2020) recommends to engage for about 150 minutes in moderate physical activity throughout the week. From the neurobiological perspective, regular engagement in physical activity can increase the dopamine level in the brain and thus provide a rewarding experience that fosters positive emotions (Knab and Lightfoot 2010). Furthermore, physical activity is an important protective factor of mental and physical health (Rebar et al. 2015). It can decrease depression symptoms (Richards et al. 2015). It contributes to life satisfaction and self-efficacy and can confer resilience in the stressful COVID-19 situation (Dwyer et al. 2020; Eime et al. 2013). In addition, physical activity provides a behavioral counterbalance to the mostly sedentary position during SMU (Bates et al. 2020; Lemenager et al. 2021).

The presented research framework allows the following considerations. Many people struggle with how to manage the changes in daily routine and how to deal with negative emotions caused by the pandemic (Evans et al. 2021). For lack of alternatives, some of them engage in online activity that is omnipresent due to social distancing and societal digitalization (Andreenkova 2020; Lemenager et al. 2021). The more an individual engages in SMU that provides positive experiences, the closer the emotional bond will be to the media, thus increasing the risk of losing control over the time spent online (Brailovskaia and Margraf 2021). An instructed and controlled reduction of SMU time could provide support in returning into the world offline and contribute to a more conscious use of the media. At the same time, an instructed and controlled increase of time spent on physical activity could be a functional alternative to experience positive emotions that some people would not come up with on their own in the stressful COVID-19 situation. The more time is spent on physical activity, the less time is available for SMU. In addition, the increasing positive emotions experienced by physical activity might weaken one’s emotional bond to the online world.

Thus, we hypothesized that a conscious and controlled reduction of time spent on SMU as well as an increase of time spent on physical activity could causally reduce negative mental health consequences of the COVID-19 situation. A combination of both interventions could increase the protective effect. Notably, both interventions can be implemented into everyday life without significant organizational efforts and costs and without violation of COVID-19 restrictions. A systematic literature search in PsycInfo, PubMed and Web of Science databases in April 2020 and again in January 2022 showed that no experimental longitudinal study – which is the only way to assess causal effects (Kraemer et al. 1997) – has so far investigated our hypothesis. Therefore, the present study aimed to investigate the influence of a controlled experimental reduction of SMU time, a controlled experimental increase of physical activity time, and a combination of both interventions on SMU, physical activity, mental health, COVID-19 burden, and smoking behavior (see Brailovskaia et al. 2020b) over a longer period of time (up to six months).

For all interventions, we hypothesized a reduction of SMU intensity (emotional bond to SMU and its integration into daily life) (Hypothesis 1a) and an increase of physical activity intensity (Hypothesis 1b). Following the dual-factor models (e.g., Keyes 2005), mental health is not only the absence of psychopathology. It consists of two interrelated but separate dimensions: positive and negative. To assess one’s mental health, both dimensions should be taken into consideration. Thus, we hypothesized that the three interventions increase life satisfaction (Hypothesis 2a) and subjective happiness (Hypothesis 2b) representing the positive dimension, and that they decrease depression symptoms (Hypothesis 2c) and addictive tendencies of SMU (Hypothesis 2d) representing the negative dimension. Moreover, we assumed that they would reduce psychological burden caused by COVID-19 (Hypothesis 3) and smoking behavior (Hypothesis 4).

Considering the lack of available causal findings (especially on a combination of both interventions), we formulated an exploratory research question to avoid speculations:

Research Question: How do the effects of the three interventions differ?

## Materials and methods

### Procedure

We designed the current study as a randomized controlled trial with three experimental groups – social media (SM) group, physical activity (PA) group, combination group – and a control group. Following Brailovskaia et al. (2020b), over a period of 14 days (= experimental manipulation period), participants of the SM group reduced their daily SMU time (= first intervention), participants of the PA group increased their daily physical activity time (= second intervention), participants of the combination group reduced their daily SMU time and increased their daily physical activity time (= third intervention), and participants of the control group did not receive specific instructions on behavioral change.

According to the World Health Organization (2020), adults aged 18–64 years are recommended to engage in at least 150 minutes of moderate physical activities per week; about 20 to 30 minutes of daily physical activity are recommended. Therefore, the PA group and the combination group increased the duration of their daily physical activity by 30 minutes over the 14 intervention days. Correspondingly, the SM group and the combination group reduced the duration of their daily SMU by 30 minutes over the 14 intervention days.

Data of the four groups were collected at six measurement time points via online surveys in German language (survey platform www.unipark.de). Participants received online links to the surveys by e-mail. Measurements took place on the day prior to the beginning of the experimental period (baseline, day 0) to assess a baseline of the variables, 1 week later (intermediate, day 7), after the 14 days of the experimental period (post, day 15), 1 month, 3 months, and 6 months after the post-measurement. This procedure enabled an investigation of the short-term and the longer-term effects of the experimental manipulation of SMU time and physical activity time. At the beginning of each survey, each participant generated an individual participant code. After the data collection, the six data sets of each participant were matched by this code.

### Participants

Data collection took place from June 2020 to December 2021. Participants were recruited by invitations displayed at public places in Germany, at German universities, and on SM such as Twitter, Facebook, and Instagram. The invitations included the following formulation: “We are investigating the relationship between social media use, physical health, and mental health. Based on the experimental condition you might be asked to change the time that you spend daily on social media use and/or on physical activity. If you would like to do this, you are welcome to participate in our investigation.” The participation was voluntary and compensated by course credits for students. People interested in participation contacted the principal investigators by e-mail. The e-mail should include their mean daily SMU time (in minutes) and their mean daily physical activity time (in minutes). The requirements for participation were daily SMU for at least 35 minutes (to prevent a complete abstinence in SM group and combination group) and engagement in physical activity for no more than one hour daily (to ensure some comparability between the participants). Performance athletes and persons with severe physical disabilities (e.g., wheelchair users) were excluded from participation. Participants had to be at least 18 years old. All requirements were included in the invitation. All participants fulfilled the requirements. The responsible Ethics Committee approved the implementation of the present study (approval number: 636). Participants were properly informed about the study and provided informed consent to participate via an online form.

All participants received an e-mail with the link to the baseline survey. At the beginning of the survey, they were randomly assigned to one of the four groups according to age and gender. The same day, the experimental groups received an e-mail including a Microsoft Word document (“daily compliance-diary”). The document included a table where participants entered daily whether they had complied with the experimental instruction (0 = *no*, 1 = *yes*). If they had not complied, they should shortly explain the reason for it. After the experimental period, they e-mailed the compliance-diary back to the principal investigators. Following Brailovskaia et al. (2020b), compliance was assessed when participants reported to have complied with the instructions on at least nine of the 14 experimental days (= two thirds). To prevent increased attention on SMU and physical activity, the control group did not receive a compliance-diary.

In addition to the diary, the e-mail of the experimental groups included group-specific instructions. Participants of the SM group and the combination group should cut down their SMU time by 30 minutes a day. To clarify the instruction, we provided each participant with a concrete maximal time that the person was allowed to spend on daily SMU over the experimental period (= daily SMU time indicated in the initial e-mail to the principal investigators minus 30 minutes). For example, if the daily SMU time was about 120 minutes, we calculated “120 minutes – 30 minutes = 90 minutes”. Therefore, the participant should engage in overall use of SM – for example Instagram, TikTok, Facebook, Twitter, WhatsApp – for no longer than 90 minutes daily.

Participants of the PA group and the combination group should increase their physical activity time by 30 minutes a day. To clarify this instruction, each participant was informed about a concrete minimal time that the person should engage in physical activity throughout a day during the experimental period (= daily physical activity time indicated in the initial e-mail to the principal investigators plus 30 minutes). The COVID-19 situation and the restrictions (lockdowns, curfews) changed over time. Therefore, we renounced to restrict our participants to specific forms of physical activity. Instead, we provided them with examples of potential forms of physical activity (e.g., jogging, cycling, swimming, Nordic walking, gymnastics, dancing, yoga; if possible, fitness studio workout, football, and basketball).

The total sample included 642 participants (all self-reported as Caucasian) who completed all surveys. Table 1 shows the demographic data of the total sample and of the four groups.

**Table 1:** Demographic data (total sample and single groups) and comparison between the groups (analysis of variance)

	Total sample	Groups				ANOVA	
		SM	PA	Combination	Control	*F*	*p*
*Age*						1.933	.123
M(SD)	26.01 (8.71)	25.11 (7.96)	26.11 (8.27)	25.51 (7.68)	27.30 (10.59)		
Min–Max	18–70	18–68	18–59	18–55	18–70		
*Gender (%)*						.683	.563
Women	78.7	80.9	80.1	74.8	78.8		
Men	21.3	19.1	19.9	25.2	21.1		
*Occupation (%)*						1.517	.209
University students	71.0	76.5	68.3	66.7	72.5		
Employees	26.0	21.6	27.3	29.6	25.6		
Unemployed	2.3	0.6	3.7	3.8	1.3		
Retired	0.6	1.2	0.6	0	0.6		
*Marital status (%)*						2.343	.072
Single	41.0	42.6	40.4	42.8	38.1		
In a relationship	47.7	50.6	45.3	50.9	43.8		
Married	11.4	6.8	14.3	6.3	18.1		

Total sample: *N* = 642, Social media (SM) group: *N* = 162, Physical activity (PA) group: *N* = 161, Combination group: *N* = 159, Control group: *N* = 160; gender: *divers* was also a response option, but no one provided this response; *M*, Mean, *SD*, Standard deviation, *Min*, Minimum, *Max*, Maximum, *ANOVA*, Analysis of Variance, *p*, significance; due to rounding the sum of the frequencies is not always 100%

#### Social media group

Overall, 182 persons were assigned to the SM group. At different stages of the investigation, 20 individuals (11%) dropped out. Thus, the SM group consisted of 162 participants who completed all surveys. Analyses of the compliance-diaries revealed a compliance rate of 93.2% (*n* = 151).

#### Physical activity group

Overall, 185 persons were assigned to the PA group. Of them, 24 individuals (13%) dropped out. Thus, the PA group consisted of 161 participants who completed all surveys. The compliance rate was 91.9% (*n* = 148).

#### Combination group

Overall, 188 persons were assigned to the combination group. Of them, 29 individuals (15.4%) dropped out. Thus, the combination group consisted of 159 participants who completed all surveys. The compliance rate was 88.1% (*n* = 140). Following Tromholt (2016) and Brailovskaia et al. (2020b), we kept non-compliers in the samples.

#### Control group

Overall, 180 persons were assigned to the control group. Of them, 20 individuals (11.1%) dropped out. Thus, the control group consisted of 160 participants who completed all surveys.

Analyses of variance (ANOVAs) showed no significant group differences of demographic variables (see Table 1), and there were no significant differences of demographic variables between individuals who dropped out and those who participated in all surveys.

A priori power analysis (G*Power program, version 3.1) indicated that at least a total sample size of *N* = 160 (*n* = 40 per group) was required for valid results (repeated measure ANOVAs, within-between factor-design; power ≥ 0.80, *α* = .05, effect size: *f* = 0.10; Mayr et al. 2007). Notably, many more people wanted to participate in the study than were provided with the a priori analysis. As we aimed to improve mental health and lifestyle, each person was included in the investigation that wanted to participate based on ethical considerations.

## Measures

### Social media use

#### Duration of SMU

Participants indicated the duration of their daily SMU (in minutes). If available, they referred to the time tracked by specific applications installed on their technical devices that they used for online activity. If not available, they estimated their usage time as accurately as possible.

#### Intensity of SMU

Intensity of SMU (emotional bond to SMU and its integration into everyday life) was assessed by a modified version of the Facebook Intensity Scale (FIS; original version: Ellison et al. 2007; German version: Brailovskaia et al. 2018a). The FIS has been validated by previous research (Brailovskaia et al. 2018a). We replaced the term “Facebook” by the term “Social Media” in the six items (e.g., “Social Media use is part of my everyday activity”). Items are rated on a 5-point Likert-type scale (1 = *disagree strongly*, 5 = *agree strongly*; current reliability: total sample at baseline: Cronbach’s *α* = .782).

#### Addictive SMU

The brief Bergen Social Media Addiction Scale (BSMAS; original version: Andreassen et al. 2016; German version: Brailovskaia et al. 2020a) measured addictive SMU. The high validity of the BSMAS has been previously described by international research on representative population samples (Brailovskaia and Margraf 2022a). Each of the six items (e.g., “Felt an urge to use Social Media more and more?”) focuses on one of the six characteristics of addictive media use (salience, tolerance, mood modification, relapse, withdrawal, conflict). Items are rated on a 5-point Likert-type scale (1 = *very rarely*, 5 = *very often*; current reliability: total sample at baseline: *α* = .837).

### Physical activity

#### Duration of physical activity

Following Fuchs et al. (2015; original German version), we asked the participants whether they had engaged in any form of physical activity during the past week (0 = *no*, 1 = *yes*). If they had done so, they were asked to name up to three activities that they engaged in. For each activity, they indicated how many times they had engaged in it and for how long (in minutes), respectively. If available, they referred to the time tracked by activity/fitness trackers. If not available, they estimated their activity time as accurately as possible. To calculate the weekly duration of physical activity, we first multiplied times and minutes (times*minutes) for each activity. Then, we summed up the product values. For participants who did not engage in any physical activity, we entered a zero. Previous research reported the validity of this way to assess the duration of physical activity (Fuchs et al. 2015).

#### Intensity of physical activity

In accordance with the assessment of SMU intensity, we used a modified version of the Facebook Intensity Scale (FIS; Ellison et al. 2007) to measure the intensity of physical activity. The term “Facebook” was replaced by “physical activity” (e.g., “Physical activity is part of my everyday activity”). The six items are rated on a 5-point Likert-type scale (1 = *disagree strongly*, 5 = *agree strongly*; current reliability: total sample at baseline: *α* = .851).

### Mental health, COVID-19 burden, and smoking behavior

#### Life satisfaction

The Satisfaction with Life Scale (SWLS; original version: Diener et al. 1985; German version: Glaesmer et al. 2011) measured global life satisfaction. The SWLS is a well-established and well-validated instrument (e.g., Bieda et al. 2017). The five items are rated on a 7-point Likert-type scale (e.g., “In most ways, my life is close to my ideal”; 1 = *strongly disagree*, 7 = *strongly agree*; current reliability: total sample at baseline: *α* = .889).

#### Subjective happiness

We used the Subjective Happiness Scale (SHS; original version: Lyubomirsky and Lepper 1999; German version: Swami et al. 2009) to assess subjective happiness. The SHS has been validated by earlier cross-national research (e.g., Bieda et al. 2017). The four items are rated on a 7-point Likert-type scale (e.g., “In general, I consider myself: 1 = *not as a very happy person*, 7 = *as a very happy person*”; current reliability: total sample at baseline: *α* = .768).

#### Depression symptoms

We assessed depression symptoms with the depression subscale of the Depression Anxiety Stress Scales 21 (DASS-21; original version: Lovibond and Lovibond 1995; German version: Nilges and Essau 2015). The DASS-21 is a well-established instrument that validity has been reported in healthy populations and in clinical patient samples (e.g., Scholten et al. 2017). The seven items (e.g., “I couldn’t seem to experience any positive feeling at all”) are rated on a 4-point Likert-type scale (0 = *did not apply to me at all*, 3 = *applied to me very much or most of the time*; current reliability: total sample at baseline: *α* = .885).

#### Psychological burden caused by COVID-19

We used the COVID-19 Burden Scale (original German version: Brailovskaia and Margraf 2021) to assess the level of psychological burden caused by COVID-19. The validity of this scale has been reported by previous international research on representative population samples (e.g., Brailovskaia et al. 2021). The six items (e.g., “I feel restricted in my everyday life”, “I am burdened by the current situation”) are rated on a 7-point Likert-type scale (1 = *I do not agree*, 7 = *I totally agree*; current reliability: total sample at baseline: *α* = .790).

#### Smoking behavior

Following available literature (Brailovskaia et al. 2020b), we asked the participants whether they regularly consume any tobacco products, such as cigarettes (0 = *no*, 1 = *yes*). Those who indicated to consume tobacco products (SM group: *n* = 28, PA group: *n* = 24, combination group: *n* = 25, control group: *n* = 20) were asked how many of them they consume daily. Earlier research confirmed the validity of this way to assess smoking behavior (Velten et al. 2014).

For all used instruments higher scores indicated higher levels of the measured variable. We assessed the variables at all six measurement time points in all groups. However, duration of daily SMU and weekly physical activity were not measured at the intermediate measurement. Notably, both variables were experimentally manipulated between baseline and post measurement in the experimental groups, while the attention of the control group should not be drawn on SMU and physical activity time. The Supplemental Material Table S1 shows the current reliability (Cronbach’s *α*) of the used instruments for each measurement time point in each group.

## Statistical analysis

Statistical analyses were conducted with the Statistical Package for the Social Sciences (SPSS 28). There were no missing data. After descriptive analyses, we ran repeated measure ANOVAs (within–between factor–design) to test potential short-term and longer-term effects (up to six measurement time points) and to compare the four investigated groups. For all variables, the assumption of sphericity (Mauchly’s test) was violated. Thus, we applied the Greenhouse–Geisser correction (ε). We used partial eta-squared (η^2^_p_) as the effect-size measure of main effects (measurement time point; group condition) and of interaction effect (measurement time point*group condition), Cohen’s d as effect-size measure of post-hoc comparisons between groups, and Cohen’s d_Repeated Measures_ as effect-size measure of post-hoc comparisons within groups. All post-hoc comparisons were Bonferroni-corrected (level of significance: *p* < .05, two-tailed).

## Results

Table 2 presents descriptive statistics of the assessed variables in the total sample at the baseline and in all groups at all six measurement time points. Considering the different manipulations in the experimental groups and the absence of any manipulation in the control group, the total sample values are no longer informative at the later measurement time points.

**Table 2:** Descriptive statistics of investigated variables (baseline to six months)

		Baseline	Intermediate	Post	One-month	Three-month	Six-month
	Group	*M (SD)*	*M (SD)*	*M (SD)*	*M (SD)*	*M (SD)*	*M (SD)*
Daily social media use time (in minutes)	Total sample	124.82 (77.69)					
	SM	131.47 (79.06)		85.40 (77.86)	100.01 (70.09)	102.88 (69.68)	94.43 (56.33)
	PA	121.58 (78.89)		102.34 (77.80)	100.32 (79.27)	94.70 (74.84)	88.91 (76.37)
	Combination	121.19 (71.64)		72.69 (50.13)	80.48 (61.43)	78.23 (56.62)	75.59 (50.34)
	Control	124.97 (81.06)		117.99 (84.21)	111.63 (76.60)	114.11 (78.78)	114.83 (84.94)
Social media use intensity	Total sample	18.87 (4.52)					
	SM	19.58 (3.99)	18.47 (4.29)	17.60 (4.63)	17.62 (4.78)	17.55 (4.57)	17.75 (4.37)
	PA	18.56 (4.81)	18.17 (4.73)	17.58 (4.87)	17.65 (4.99)	17.52 (4.90)	17.95 (4.42)
	Combination	18.96 (4.81)	16.97 (5.02)	16.52 (5.09)	16.55 (5.06)	15.98 (5.31)	16.27 (5.33)
	Control	18.39 (4.61)	18.11 (4.39)	18.04 (4.77)	18.66 (5.04)	18.66 (5.54)	18.79 (5.24)
Weekly physical activity time (in minutes)	Total sample	123.42 (133.26)					
	SM	125.55 (128.35)		136.97 (156.90)	139.50 (192.95)	181.53 (234.15)	151.81 (183.39)
	PA	117.80 (131.52)		294.41 (219.52)	193.82 (252.91)	195.86 (247.33)	158.20 (201.06)
	Combination	125.33 (137.07)		290.41 (188.63)	219.25 (222.61)	212.86 (234.95)	224.13 (229.95)
	Control	125.01 (137.07)		134.23 (172.39)	121.70 (165.67)	141.25 (206.22)	145.12 (247.60)
Physical activity intensity	Total sample	42.91 (6.34)					
	SM	19.48 (5.93)	19.24 (5.93)	19.32 (5.90)	19.73 (5.52)	19.98 (5.48)	19.82 (5.44)
	PA	19.39 (5.24)	20.96 (4.71)	21.03 (4.90)	20.53 (5.31)	20.52 (5.10)	20.46 (5.35)
	Combination	19.72 (5.26)	20.88 (4.72)	21.01 (4.82)	21.12 (4.81)	21.16 (4.99)	22.05 (4.28)
	Control	19.31 (5.84)	19.49 (5.49)	19.24 (5.91)	19.51 (5.61)	19.28 (5.71)	19.48 (5.37)
Life satisfaction	Total sample	25.34 (5.95)					
	SM	25.20 (5.94)	25.80 (5.60)	26.43 (4.91)	26.33 (5.25)	26.41 (5.47)	26.06 (5.62)
	PA	25.32 (5.88)	25.69 (5.93)	26.40 (6.15)	25.91 (6.01)	25.88 (6.16)	25.93 (6.34)
	Combination	25.55 (5.85)	25.95 (5.57)	27.36 (4.85)	27.12 (5.18)	26.97 (5.28)	27.16 (4.73)
	Control	25.31 (6.19)	25.23 (5.77)	25.26 (6.50)	25.17 (6.49)	25.23 (6.39)	25.35 (5.96)
Subjective happiness	Total sample	18.98 (4.47)					
	SM	18.83 (4.45)	18.90 (4.24)	19.17 (4.37)	19.74 (4.11)	19.41 (4.09)	19.66 (4.35)
	PA	18.74 (4.28)	19.19 (4.19)	19.45 (4.43)	19.36 (4.59)	19.26 (4.20)	19.43 (4.39)
	Combination	18.97 (4.62)	19.60 (4.54)	20.02 (4.10)	19.94 (4.55)	20.69 (3.82)	21.13 (3.44)
	Control	19.38 (4.53)	19.18 (4.34)	19.01 (4.53)	19.19 (4.70)	19.30 (4.75)	19.33 (4.64)
Depression symptoms	Total sample	4.73 (4.43)					
	SM	5.02 (4.63)	4.31 (4.15)	4.16 (4.11)	4.27 (4.06)	4.14 (4.29)	4.39 (4.27)
	PA	4.92 (4.09)	4.30 (3.89)	3.75 (3.92)	4.01 (4.14)	4.07 (3.99)	4.01 (4.24)
	Combination	4.63 (4.37)	3.08 (3.22)	3.18 (3.40)	3.05 (3.35)	2.88 (3.27)	2.89 (3.18)
	Control	4.34 (4.61)	4.29 (4.29)	4.44 (4.72)	4.28 (4.49)	4.26 (4.43)	4.44 (4.49)
Addictive social media use	Total sample	13.21 (5.07)					
	SM	13.86 (5.14)	13.60 (5.31)	13.16 (5.33)	12.72 (4.99)	12.84 (5.22)	13.32 (5.36)
	PA	12.97 (5.04)	13.35 (5.55)	13.11 (5.48)	12.70 (5.64)	12.21 (5.36)	12.46 (5.38)
	Combination	13.24 (5.17)	12.34 (5.07)	11.47 (4.64)	11.39 (4.61)	11.29 (4.75)	11.56 (5.15)
	Control	12.76 (4.92)	12.96 (5.26)	13.04 (5.48)	12.96 (5.40)	13.07 (4.94)	13.36 (5.08)
COVID-19 burden	Total sample	24.54 (6.73)					
	SM	24.10 (7.20)	23.59 (7.12)	23.33 (7.07)	21.85 (7.61)	21.41 (8.15)	21.86 (6.69)
	PA	25.25 (6.41)	24.60 (6.59)	23.68 (6.98)	22.58 (6.90)	22.27 (7.30)	22.01 (7.26)
	Combination	23.62 (6.85)	23.13 (6.57)	22.66 (7.80)	21.02 (7.76)	21.07 (7.87)	20.74 (7.30)
	Control	25.19 (6.35)	24.52 (6.78)	24.34 (6.83)	24.96 (8.02)	24.70 (8.06)	24.49 (6.97)
Smoking behavior (number of daily consumed tobacco products)	Total sample	6.45 (6.58)					
	SM	6.71 (7.79)	6.89 (7.68)	4.64 (5.65)	4.07 (5.00)	3.89 (4.69)	3.79 (4.83)
	PA	5.50 (5.83)	4.08 (4.65)	4.38 (4.75)	4.04 (4.63)	4.08 (4.56)	4.92 (5.59)
	Combination	6.48 (5.30)	6.40 (5.47)	6.24 (5.36)	5.80 (5.59)	4.52 (4.66)	3.84 (4.01)
	Control	7.20 (7.35)	6.90 (8.60)	6.55 (8.66)	6.60 (7.67)	6.30 (6.48)	6.50 (7.56)

Total sample: *N* = 642, Social media (SM) group: *N* = 162, Physical activity (PA) group: *N* = 161, Combination group: *N* = 159, Control group: *N* = 160; exception: smoking behavior: SM group: *n* = 28, PA group: *n* = 24, Combination group: *n* = 25, Control group: *n* = 20; Baseline to six-month = measurement time points; *M*, Mean; *SD*, Standard Deviation

**Fig. 1:**
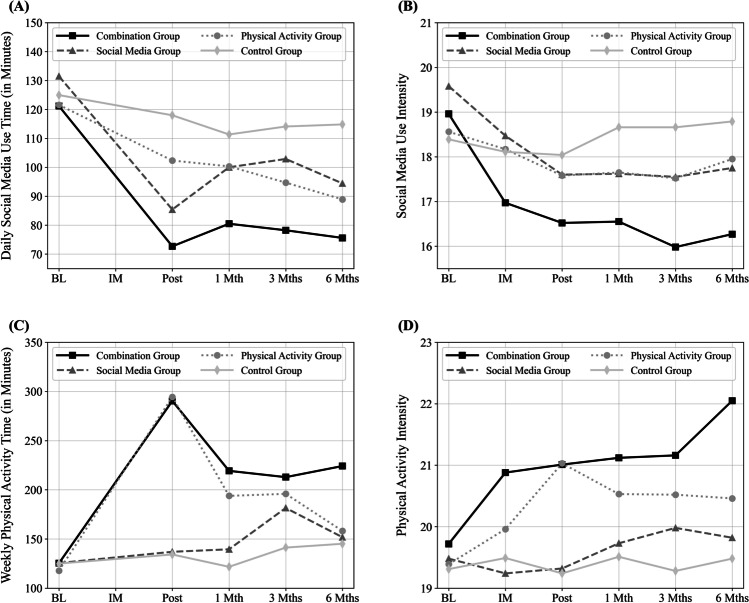
Results of repeated measure analyses of variance (ANOVAs) for time and intensity of social media use and of physical activity: (**A**) daily social media use time (in minutes), (**B**) intensity of social media use, (**C**) weekly physical activity (in minutes), (**D**) intensity of physical activity (Social media group: *N* = 162, Physical activity group: *N* = 161, Combination group: *N* = 159, Control group: *N* = 160; BL to 6 Mths (Months) = measurement time points)

**Fig. 2:**
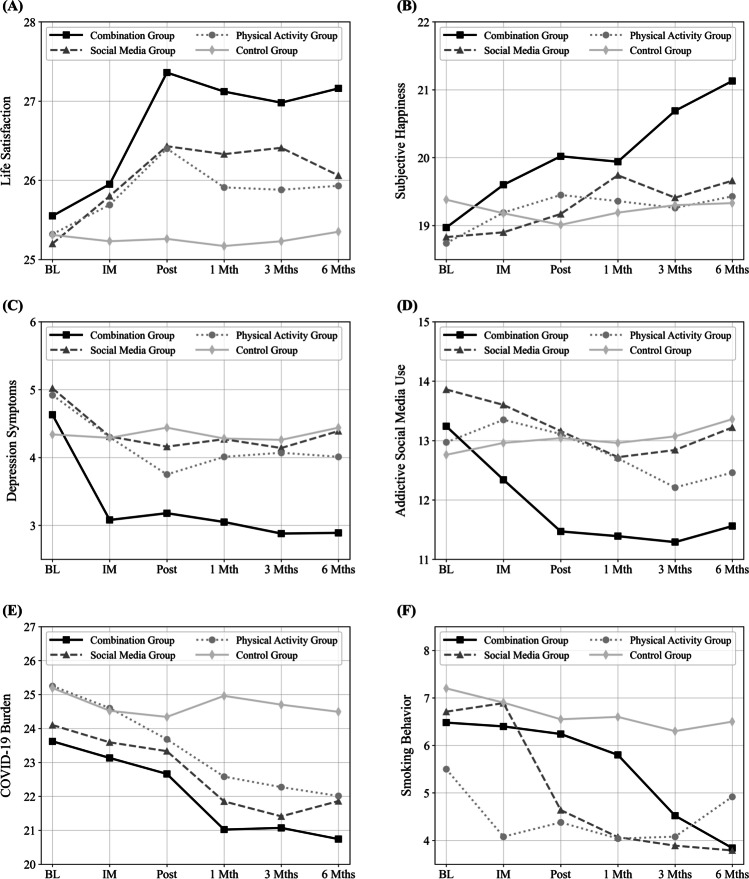
Results of repeated measure analyses of variance (ANOVAs) for variables of mental health and lifestyle: (**A**) life satisfaction, (**B**) subjective happiness, (**C**) depression symptoms, (**D**) addictive social media use, (**E**) COVID-19 burden, (**F**) smoking behavior (Social media group: *N* = 162, Physical activity group: *N* = 161, Combination group: *N* = 159, Control group: *N* = 160; exception: smoking behavior: Social media group: *n* = 28, Physical activity group: *n* = 24, Combination group: *n* = 25, Control group: *n* = 20; BL to 6 Mths (Months) = measurement time points)

Figure 1 (time and intensity of SMU and of physical activity) and Fig. 2 (variables of mental health, COVID-19 burden, smoking behavior) visualize results of the ANOVAs.

Supplemental Material Table S2 shows significant results of pairwise comparisons within groups, Supplemental Material Table S3 summarizes significant results of pairwise comparisons between groups. As there were no significant group differences at baseline, Supplemental Material Table S3 starts with intermediate measurement.

For time spent daily on SMU, the ANOVA revealed a significant main effect for measurement time point, *F*(3.46, 2208.33) = 62.708, *p* < .001, η^2^_p_ = .089, and for group condition, *F*(3, 638) = 6.651, *p* < .001, η^2^_p_ = .030, and a significant interaction effect, *F*(10.38, 2208.33) = 6.115, *p* < .001, η^2^_p_ = .028. Pairwise comparisons showed a significant decrease of SMU time in all experimental groups. Most within-group effects were stronger in the combination group than in the other groups (see Supplemental Material Table S2). SMU time was significantly lower in the SM group (post, 6-month), the PA group (6-month), and the combination group (post, 1-month, 3-month, 6-month) than in the control group. Also, SMU time was significantly lower in the combination group than in the PA group (post) and in the SM group (3-month) (see Supplemental Material Table S3).

For intensity of SMU, the ANOVA provided a significant main effect for measurement time point, *F*(4.20, 2678.34) = 27.392, *p* < .001, η^2^_p_ = .041, and for group condition, *F*(3, 638) = 4.154, *p* = .006, η^2^_p_ = .019, and a significant interaction effect, *F*(12.59, 2678.34) = 6.734, *p* < .001, η^2^_p_ = .031. Pairwise comparisons revealed a significant decrease of SMU intensity in the SM group and the combination group over the measurement time points. In the PA group, use intensity was lower at post-intervention and at 3-month measurement than at baseline only (see Supplemental Material Table S2). SMU intensity was significantly lower in the combination group than in the SM group (intermediate, 3-month, 6-month), the PA group (3-month, 6-month), and the control group (post, 1-month, 3-month, 6-month) (see Supplemental Material Table S3).

For time spent weekly on physical activity, the ANOVA revealed a significant main effect for measurement time point, *F*(3.64, 2319.63) = 26.567, *p* < .001, η^2^_p_ = .040, and for group condition, *F*(3, 638) = 12.163, *p* < .001, η^2^_p_ = .054, and a significant interaction effect, *F*(10.91, 2319.63) = 8.427, *p* < .001, η^2^_p_ = .038. Pairwise comparisons provided a significant increase of physical activity time in the combination group. The main increase was between baseline and post-intervention measurement. After post-intervention measurement, its level decreased. But it remained significantly higher than the baseline level even at the 6-month measurement. There was a similar pattern in the PA group. However, the decrease of physical activity time after post-intervention measurement resulted in no significant difference between the baseline and 6-month measurement. In the SM group, physical activity time increased between the baseline and 3-month measurement (see Supplemental Material Table S2). Physical activity time was significantly higher in the combination group than in the SM group (post, 1-month, 6-month), the PA group (6-month), and the control group (post, 1-month, 3-month, 6-month). Also, it was significantly higher in the PA group than in the SM group (post) and the control group (post, 1-month) (see Supplemental Material Table S3).

For intensity of physical activity, the ANOVA showed a significant main effect for measurement time point, *F*(4.17, 2662.56) = 11.17, *p* < .001, η^2^_p_ = .017, and for group condition, *F*(3, 638) = 3.919, *p* = .009, η^2^_p_ = .018, and a significant interaction effect, *F*(12.52, 2662.56) = 4.742, *p* < .001, η^2^_p_ = .022. As shown by pairwise comparisons, the intensity of physical activity significantly increased in the PA group and in the combination group after the baseline measurement. After the initial increase, there was a further significant increase of the intensity at the 6-month measurement in the combination group only (see Supplemental Material Table S2). Physical activity intensity was significantly higher in the combination group than in the SM group (intermediate, post, 6-month), the PA group (6-month), and the control group (post, 1-month, 3-month, 6-month). Also, it was significantly higher in the PA group than in the SM group (intermediate, post) and the control group (post) (see Supplemental Material Table S3).

For life satisfaction, the ANOVA revealed a significant main effect for measurement time point, *F*(4.09, 2606.02) = 11.970, *p* < .001, η^2^_p_ = .018, a non-significant main effect for group condition, *F*(3, 638) = 2.044, *p* = .107, and a significant interaction effect, *F*(12.25, 2606.02) = 2.427, *p* = .004, η^2^_p_ = .011. Following pairwise comparisons, life satisfaction significantly increased in the combination group. It also increased in the SM group and in the PA group, but the effects were smaller and less stable than in the combination group in the longer-term (see Supplemental Material Table S2). Life satisfaction was significantly higher in the combination group than in the control group (post, 1-month, 3-month, 6-month) (see Supplemental Material Table S3).

For subjective happiness, the ANOVA showed a significant main effect for measurement time point, *F*(4.54, 2898.90) = 11.766, *p* < .001, η^2^_p_ = .018, a non-significant main effect for group condition, *F*(3, 638) = 1.848, *p* = .137, and a significant interaction effect, *F*(13.63, 2898.90) = 3.728, *p* < .001, η^2^_p_ = .017. Pairwise comparisons revealed a significant increase of happiness in the combination group. It also significantly increased in the SM group, but the effects were smaller and less stable than in the combination group in the longer-term (see Supplemental Material Table S2). Happiness was significantly higher in the combination group than in the other three groups (3-month, 6-month) (see Supplemental Material Table S3).

For depressive symptoms, the ANOVA provided a significant main effect for measurement time point, *F*(4.50, 2868.58) = 12.729, *p* < .001, η^2^_p_ = .020, and for group condition, *F*(3, 638) = 3.543, *p* = .014, η^2^_p_ = .016, and a significant interaction effect, *F*(13.49, 2868.58) = 2.480, *p* = .002, η^2^_p_ = .012. Pairwise comparisons revealed a significant decrease of depression symptoms in the combination group. They also significantly decreased in the SM group and in the PA group. But the effects were smaller and less stable than in the combination group in the longer-term (see Supplemental Material Table S2). Depression symptoms were significantly higher in the combination group than in the SM group (intermediate, 1-month, 3-month, 6-month), the PA group (intermediate, 3-month), and the control group (intermediate, post, 1-month, 3-month, 6-month) (see Supplemental Material Table S3).

For addictive SMU, the ANOVA showed a significant main effect for measurement time point, *F*(4.41, 2814.62) = 11.272, *p* < .001, η^2^_p_ = .017, and for group condition, *F*(3, 638) = 2.682, *p* = .046, η^2^_p_ = .012, and a significant interaction effect, *F*(13.24, 2814.62) = 4.436, *p* < .001, η^2^_p_ = .020. Pairwise comparisons showed a significant decrease of addictive SMU in the combination group. There was also a significant decrease in the SM group and in the PA group, but the effects were less stable than in the combination group in the longer-term (see Supplemental Material Table S2). Addictive SMU was significantly lower in the combination group than in the SM group (post, 3-month, 6-month), the PA group (post), and the control group (post, 1-month, 3-month, 6-month) (see Supplemental Material Table S3).

For psychological burden by COVID-19, the ANOVA provided a significant main effect for measurement time point, *F*(3.64, 2321.91) = 24.787, *p* < .001, η^2^_p_ = .037, and for group condition, *F*(3, 638) = 6.289, *p* < .001, η^2^_p_ = .029, and a significant interaction effect, *F*(10.92, 2321.91) = 2.733, *p* = .002, η^2^_p_ = .013. Pairwise comparisons revealed a significant decrease of COVID-19 burden in the experimental groups (see Supplemental Material Table S2). COVID-19 burden was significantly lower in the experimental groups than in the control group (1-month, 3-month, 6-month) with the strongest effects in the combination group (see Supplemental Material Table S3).

For smoking behavior, the ANOVA provided a significant main effect for measurement time point, *F*(3.00, 278.74) = 6.927, *p* < .001, η^2^_p_ = .069, a non-significant effect for group condition, *F*(3, 93) = .630, *p* = .597, and a significant interaction effect, *F*(8.99, 278.74) = 1.959, *p* = .044, η^2^_p_ = .059. Pairwise comparisons showed a significant decrease of smoking behavior in the SM group. There was also a decrease in the combination group, but it was not significant, which at least partly could be due to the fact that only a small percentage of the group reported smoking behavior (see Supplemental Material Table S2).

## Discussion

Each year, large sums of money are spent on complex intervention programs for mental health protection (e.g., Mental Health Foundation 2016; Leonhardt 2021; CBS 2015). The pandemic is accompanied by an increase of psychological burden and a decrease of mental health (Bueno-Notivol et al. 2020). Following available literature (e.g., Shakya and Christakis 2017), excessive SMU in everyday life that increased since the pandemic outbreak (Luo et al. 2021) can negatively impact mental health.

The experimental longitudinal design of the current study allows causal conclusions. Our findings show that a conscious and controlled reduction of SMU time as well as an increase of physical activity time can causally improve mental health and decrease the COVID-19 burden for up to six months. The combination of both interventions provided the strongest and most stable positive effect on most of the investigated variables (see Research Question).

Our interventions contributed to a decrease of time spent on SMU after the experimental period. Six months later, the participants had reduced their daily initial SM time by about 37 minutes in the SM group, by about 33 minutes in the PA group, and by about 46 minutes in the combination group. The intensity of the use – emotional bond to it and its integration in everyday life – decreased in the experimental groups (confirmation of Hypothesis 1a). Notably, this effect was weaker in the PA group than in the other groups. Furthermore, all interventions resulted in an increase of time spent on physical activity after the experimental period. Six months later, our participants had enhanced their initial weekly physical activity time for 26 minutes in the SM group, for 40 minutes in the PA group, and for 1 hour 39 minutes in the combination group. Interestingly, there was also an increase of physical activity time for 20 minutes in the control group that, however, was not significant. The intensity of physical activity increased in the PA group and in the combination group, but not in the SM group (partial confirmation of Hypothesis 1b). In the longer-term, the effect was stronger in the combination group than in the PA group.

Overall, the findings on Hypothesis 1 reveal that to weaken the emotional bond to SMU longitudinally, it is not enough to only decrease SMU time or to only provide people with healthier behavioral alternatives such as physical activity. Also, to foster the bond to physical activity longitudinally, it is not enough to only enhance the physical activity time. A combination of both interventions is important for a successful longitudinal reduction of the role SMU plays in one’s everyday life, while the meaning of physical activity increases. The not significant finding in the control group underlines this conclusion. It seems that just answering the surveys could foster the intention to engage in a healthier lifestyle. However, the effect is not strong enough for a significant change in everyday life.

Moreover, our interventions influenced both dimensions of mental health (Keyes 2005). Most effects were remarkably stronger in the combination group than in the distinct experimental groups. Following previous research, life satisfaction and subjective happiness are negatively linked to SMU (Shakya and Christakis 2017; Twenge 2019). Their relationship with physical activity is positive (Maher et al. 2015; Richards et al. 2015). In line with these findings, life satisfaction increased in all experimental groups (confirmation of Hypothesis 2a). Happiness increased in the SM group and in the combination group (partial confirmation of Hypothesis 2b). Intensive SMU can contribute to depression symptoms (Brailovskaia et al. 2019), while physical activity reduces them (Rebar et al. 2015). In the present study, depression symptoms decreased in all experimental groups (confirmation of Hypothesis 2c). Time spent online can foster an emotional bond to the SMU that predisposes addictive tendencies (Andreassen et al. 2016). Physical activity has been described to reduce addictive SMU (Brailovskaia et al. 2018b). In line with this knowledge, addictive SMU decreased in the experimental groups (confirmation of Hypothesis 2d). Furthermore, all introduced interventions decreased the COVID-19 burden (confirmation of Hypothesis 3).

The following considerations can at least partly explain our findings. Humans are social beings and social interaction is part of their essential needs (Dahrendorf 2010). The engagement in social interaction reinforces positive emotions and decreases negative ones, which is important for the maintenance of mental health. Positive experiences often foster further interaction to increase the gratification (Gloria and Steinhardt 2016). With the development of mobile technical devices, SMU overcame temporal and spatial borders. This opened new opportunities for social interaction (Sun and Zhang 2020).

Following the Interaction of Person-Affect-Cognition-Execution (I-PACE) model for addictive behavior (Brand et al. 2016), due to an interaction between environmental and person-specific factors, gratification experienced during SMU can result in developing a habit of prolonged online activity. As a consequence, the person tends to less reflective behavior and to more impulsive responses, which implies excessive SMU in different situations (Brand et al. 2019). Excessive SMU can foster addictive tendencies, depression symptoms, and a decrease of life satisfaction in the longer-term (Sun and Zhang 2020). The pandemic limited offline experiences and face-to-face contacts and thus SMU became a crucial element of everyday life (Lemenager et al. 2021) that might reinforce the processes described by the I-PACE model.

Previous research showed that a reduction of SMU time can improve mental health (Hunt et al. 2018). Engagement in physical activity is positively linked to neuronal processes of gratification (Knab and Lightfoot 2010). Furthermore, small personal achievements during physical activity such as an increase of one’s jogging speed can foster self-efficacy and positive emotions that are significant for mental health (McAuley et al. 2006). The present study combined the reduction of SMU time with the increase of physical activity time for the first time. By answering the six surveys, our participants reflected consciously on their SMU, lifestyle, and mental health. Some of them did this for the first time as described in the compliance-diaries. The cognitive dealing with own SMU and its potential consequences could be the first step to a more conscious handling of one’s online activity (see King et al. 2012). However, the not significant results of the control group show that this might not be enough to break down strong SMU habits that are based on impulsive reactions. By the controlled SMU reduction, we supported the participants in doing so. However, the experience of gratification is an important mechanism that fosters impulsive SMU. The awareness of alternative behavior that could also provide gratification is lacking (Brand et al. 2019). By the combination of the SMU reduction with an increase of conscious physical activity, we provided the participants with a functional alternative for gratification experience instead of mere restrictions. This is of specific importance during the COVID-19 situation that interrupted daily routine and limited the options for gratification (Evans et al. 2021). Thus, by increasing cognitive awareness of one’s SMU and breaking down impulsive SMU we reduced a risk factor for mental health. At the same time, we introduced a protective factor (physical activity as a source of gratification) in the combination group. This might explain the strong and stable positive effects.

In line with Brailovskaia et al. (2020b) who focused on Facebook use, we found a reduction of smoking behavior in the SM group: from initially about 6 to 7 tobacco products daily to 3 to 4 products six months after the experimental period. The numerical decrease in the combination group was not statistically significant. The not significant findings in the PA group reveal that to attract one’s attention to physical activity as a component of a healthy lifestyle is not sufficient to influence smoking behavior (partial confirmation of Hypothesis 4). We assume that the increase of cognitive awareness of one’s SMU in combination with a breaking down of impulsive online activity might enhance the participants’ awareness of other unhealthy behavior and health risks, which might foster a striving for a healthier lifestyle. As a consequence, they could consciously reduce their smoking behavior. Furthermore, some people tend to smoke when they are stressed (Kassel et al. 2003). Thus, the improvement of the mental health variables and the decrease of COVID-19 burden might also contribute to reduced smoking behavior. Some people engage in SMU via smartphone in the so-called smoker breaks – taking a break during the performance of a specific task to have a smoke (Delaney et al. 2018). It could be that the reduction of SMU time might also contribute to a reduction of such breaks.

Despite the potential benefits of SMU especially during the pandemic, the present causal findings reveal that it is important to consciously reduce the SMU from time to time to protect mental health, to reduce the COVID-19 burden, and to foster a healthier lifestyle (more physical activity, less SMU, less smoking behavior) in the longer-term. The protective effect can be enhanced by a combination of SMU reduction with a conscious increase of physical activity time. Both strategies can be implemented in one’s everyday life without expensive time-consuming efforts in the COVID-19 situation by online instructions. They can also serve as an addition in psychotherapeutic treatment of, for example, depression symptoms in the clinical setting. Also, they can be incorporated into general mental health prevention programs or into specific anti-smoking campaigns to increase their effectivity.

The present study has some limitations. First, the mostly female, young, and well-educated composition of our sample that was mostly recruited from the same region in Germany limits the findings’ generalizability. Our results should be replicated in more population representative groups. Second, the COVID-19 incidence rates and introduced restrictions differ between and within countries at different points of time (World Health Organization 2021). Our data collection took place only in Germany during a limited period of time. Its replication in other countries and at other points in time is desirable. In addition, it could be assessed whether participants’ physical health is affected by the pandemic. This could influence the level of their physical activity. Also, the enhanced physical activity in our experimental groups could influence the participants’ physical health state. As we did not assess variables of physical health, future experimental studies should investigate this issue to prevent speculations. Third, we used self-report measures for data collection that are prone to social desirability, same-source bias, and distortions of perception (Conway and Lance 2010; Musch et al. 2002). Thus, our findings should be interpreted with caution. The compliance-diaries provided us with information about participants’ SMU and physical activity at least partly. Furthermore, we asked the participants to reveal the SMU time and physical activity time tracked by specific applications and devices if available. Future studies should assess the physical activity time of all participants by activity/fitness trackers. SMU time could be tracked by specific applications that participants can install on their technical devices. Hereby, it should be considered that different technical devices (e.g., laptop, tablet, smartphone) can be used for online activity. The overall usage time must be summed up. Fourth, previous research showed that active (e.g., uploading of content) and passive (e.g., browsing of updates posted by others) SMU can have various effects on mental health. For example, passive use can foster the feeling of envy that contributes to depression symptoms (e.g., Verduyn et al. 2015). Active use is closely associated with the experience of flow that contributes to addictive use tendencies (Brailovskaia and Margraf 2022b). Brailovskaia et al. (2020b) described a reduction of both forms of use after a controlled reduction of daily time spent on Facebook use. In the present study, we did not assess which forms of SMU the participants changed during the reduction period of SMU time. To gain further insights in the mechanisms that could explain our results, future studies should focus on the different forms of SMU and their potential changes during and after the experimental period. Fifth, due to the unpredictability of the COVID-19 situation, we provided only examples of potential forms of physical activity that the participants could engage in. A more standardized instruction on the form of physical activity in future studies could increase the comparability within the sample. Furthermore, our focus was on the quantity of physical activity. Future research should focus more precisely on the form/quality of physical activity that participants change during and after the experimental period for a better understanding of the mechanisms that underly the positive effects of the experimental manipulation.

SMU prevents isolation during the pandemic and allows a rapid spread of information. However, our causal findings indicate that its moderate reduction in combination with a moderate increase of physical activity could be an effective and cost-efficient strategy to protect mental health. From time to time, it is important to consciously limit one’s online accessibility and to go back to the human roots – that evolutionary imply a physically active lifestyle (Bouchard et al. 2012) – to stay happy and healthy in the age of digitalization.

## Supplementary information

ESM 1 (DOCX 72 kb)

ESM 2 (DOCX 93 kb)

ESM 3 (DOCX 73 kb)

## Data Availability

The dataset and further material analysed during the current study will be available from the corresponding author on reasonable request.

## Code availability (software application or custom code)

Statistical analyses were conducted using SPSS 28. The used statistical codes will be available from the corresponding author on reasonable request.

## References

[CR1] Andreassen CS, Billieux J, Griffiths MD (2016). The relationship between addictive use of social media and video games and symptoms of psychiatric disorders: a large-scale cross-sectional study. Psychol Addict Behav.

[CR2] Andreenkova AV (2020). Digitalization of social contacts among university students in Russia during Covid-19. Monitoring Obshchestvennogo Mneniya: Ekonomicheskie i Sotsial'nye Peremeny.

[CR3] Bates LC, Zieff G, Stanford K (2020). COVID-19 impact on behaviors across the 24-hour day in children and adolescents: physical activity, sedentary behavior, and sleep. Children.

[CR4] Bieda A, Hirschfeld G, Schönfeld P (2017). Universal happiness? Cross-cultural measurement invariance of scales assessing positive mental health. Psychol Assess.

[CR5] Bouchard C, Blair SN, Haskell WL (2012). Physical activity and health.

[CR6] Brailovskaia J, Margraf J (2021). The relationship between burden caused by coronavirus (Covid-19), addictive social media use, sense of control and anxiety. Comput Hum Behav.

[CR7] Brailovskaia J, Margraf J (2022a) Addictive social media use during Covid-19 outbreak: validation of the Bergen social media addiction scale (BSMAS) and investigation of protective factors in nine countries. Curr Psychol. 10.1007/s12144-022-03182-z 10.1007/s12144-022-03182-z PMC9122809 35615694

[CR8] Brailovskaia J, Margraf J (2022). The relationship between active and passive Facebook use, Facebook flow, depression symptoms and Facebook addiction: a three-month investigation. J Affective Disorders Reports.

[CR9] Brailovskaia J, Rohmann E, Bierhoff H-W (2018). The brave blue world: Facebook flow and Facebook addiction disorder (FAD). PLoS One.

[CR10] Brailovskaia J, Teismann T, Margraf J (2018). Physical activity mediates the association between daily stress and Facebook addiction disorder (FAD) – a longitudinal approach among German students. Comput Hum Behav.

[CR11] Brailovskaia J, Rohmann E, Bierhoff H-W (2019). Relationships between addictive Facebook use, depressiveness, insomnia, and positive mental health in an inpatient sample: a German longitudinal study. J Behav Addict.

[CR12] Brailovskaia J, Schillack H, Margraf J (2020). Tell me why are you using social media (SM)! Relationship between reasons for use of SM, SM flow, daily stress, depression, anxiety, and addictive SM use–an exploratory investigation of young adults in Germany. Comput Hum Behav.

[CR13] Brailovskaia J, Ströse F, Schillack H (2020). Less Facebook use–more well-being and a healthier lifestyle? An experimental intervention study. Comput Hum Behav.

[CR14] Brailovskaia J, Schneider S, Margraf J (2021). To vaccinate or not to vaccinate!? Predictors of willingness to receive Covid-19 vaccination in Europe, the U.S., and China. PLoS One.

[CR15] Brand M, Young KS, Laier C (2016). Integrating psychological and neurobiological considerations regarding the development and maintenance of specific internet-use disorders: an interaction of person-affect-cognition-execution (I-PACE) model. Neurosci Biobehav Rev.

[CR16] Brand M, Wegmann E, Stark R (2019). The interaction of person-affect-cognition-execution (I-PACE) model for addictive behaviors: update, generalization to addictive behaviors beyond internet-use disorders, and specification of the process character of addictive behaviors. Neurosci Biobehav Rev.

[CR17] Bueno-Notivol J, Gracia-García P, Olaya B (2020). Prevalence of depression during the COVID-19 outbreak: a meta-analysis of community-based studies. Int J Clin Health Psychol.

[CR18] CBS (2015) Relatively high budget for mental health care services. Available at: https://www.cbs.nl/en-gb/news/2015/49/relatively-high-budget-for-mental-health-care-services. Accessed 5 July 2022

[CR19] Conway JM, Lance CE (2010). What reviewers should expect from authors regarding common method bias in organizational research. J Bus Psychol.

[CR20] Dahrendorf R (2010). Homo Sociologicus. Ein Versuch zur Geschichte, Bedeutung und Kritik der sozialen Rolle.

[CR21] Delaney H, MacGregor A, Amos A (2018). “Tell them you smoke, you’ll get more breaks”: a qualitative study of occupational and social contexts of young adult smoking in Scotland. BMJ Open.

[CR22] Diener E, Emmons RA, Larsen RJ (1985). The satisfaction with life scale. J Pers Assess.

[CR23] Dwyer MJ, Pasini M, De Dominicis S (2020). Physical activity: benefits and challenges during the COVID-19 pandemic. Scand J Med Sci Sports.

[CR24] Eime RM, Young JA, Harvey JT (2013). A systematic review of the psychological and social benefits of participation in sport for adults: informing development of a conceptual model of health through sport. Int J Behav Nutr Phys Act.

[CR25] Ellison NB, Steinfield C, Lampe C (2007). The benefits of Facebook “friends:” social capital and college students’ use of online social network sites. J Comput-Mediat Commun.

[CR26] Evans S, Alkan E, Bhangoo JK (2021). Effects of the COVID-19 lockdown on mental health, wellbeing, sleep, and alcohol use in a UK student sample. Psychiatry Res.

[CR27] Fuchs R, Klaperski S, Gerber M (2015). Messung der Bewegungs-und Sportaktivität mit dem BSA-Fragebogen. Zeitschrift für Gesundheitspsychologie.

[CR28] Glaesmer H, Grande G, Braehler E (2011). The German version of the satisfaction with life scale (SWLS): psychometric properties, validity, and population-based norms. Eur J Psychol Assess.

[CR29] Gloria CT, Steinhardt MA (2016). Relationships among positive emotions, coping, resilience and mental health. Stress Health.

[CR30] Gray J, Rumpe B (2015). Models for digitalization. Software Syst Model.

[CR31] Hunt MG, Marx R, Lipson C (2018). No more FOMO: limiting social media decreases loneliness and depression. J Soc Clin Psychol.

[CR32] Kassel JD, Stroud LR, Paronis CA (2003). Smoking, stress, and negative affect: correlation, causation, and context across stages of smoking. Psychol Bull.

[CR33] Keyes CLM (2005). Mental illness and/or mental health? Investigating axioms of the complete state model of health. J Consult Clin Psychol.

[CR34] King DL, Delfabbro PH, Griffiths MD (2012). Cognitive-behavioral approaches to outpatient treatment of internet addiction in children and adolescents. J Clin Psychol.

[CR35] Knab AM, Lightfoot JT (2010). Does the difference between physically active and couch potato lie in the dopamine system?. Int J Biol Sci.

[CR36] Kraemer HC, Kazdin AE, Offord DR (1997). Coming to terms with the terms of risk. Arch Gen Psychiatry.

[CR37] Lemenager T, Neissner M, Koopmann A (2021). COVID-19 lockdown restrictions and online media consumption in Germany. Int J Environ Res Public Health.

[CR38] Leonhardt M (2021) What you need to know about the cost and accessibility of mental health care in America. Available at: https://www.cnbc.com/2021/05/10/cost-and-accessibility-of-mental-health-care-in-america.html. Accessed 5 July 2022

[CR39] Lovibond PF, Lovibond SH (1995). The structure of negative emotional states: comparison of the depression anxiety stress scales (DASS) with the Beck depression and anxiety inventories. Behav Res Ther.

[CR40] Luo T, Chen W, Liao Y (2021). Social media use in China before and during COVID-19: preliminary results from an online retrospective survey. J Psychiatr Res.

[CR41] Lyubomirsky S, Lepper HS (1999). A measure of subjective happiness: preliminary reliability and construct validation. Soc Indic Res.

[CR42] Maher JP, Pincus AL, Ram N (2015). Daily physical activity and life satisfaction across adulthood. Dev Psychol.

[CR43] Mano R (2020). Social media and resilience in the COVID-19 crisis. Adv Appl Sociol.

[CR44] Mayr S, Erdfelder E, Buchner A (2007). A short tutorial of GPower. Tutor Quantitative Methods Psychol.

[CR45] McAuley E, Konopack JF, Motl RW (2006). Physical activity and quality of life in older adults: influence of health status and self-efficacy. Ann Behav Med.

[CR46] Mental Health Foundation (2016). Fundamental facts about mental health 2016.

[CR47] Musch J, Brockhaus R, Bröder A (2002). An inventory for the assessment of two factors of social desirability. Diagnostica.

[CR48] Nagel L (2020). The influence of the COVID-19 pandemic on the digital transformation of work. Int J Sociol Soc Policy.

[CR49] Nilges P, Essau C (2015). Die Depressions-Angst-Stress-Skalen. Schmerz.

[CR50] Ornell F, Schuch JB, Sordi AO (2020). “Pandemic fear” and COVID-19: mental health burden and strategies. Brazilian J Psychiat.

[CR51] Parviainen P, Tihinen M, Kääriäinen J (2017). Tackling the digitalization challenge: how to benefit from digitalization in practice. Int J Inf Syst Proj Manag.

[CR52] Rebar AL, Stanton R, Geard D (2015). A meta-meta-analysis of the effect of physical activity on depression and anxiety in non-clinical adult populations. Health Psychol Rev.

[CR53] Richards J, Jiang X, Kelly P (2015). Don't worry, be happy: cross-sectional associations between physical activity and happiness in 15 European countries. BMC Public Health.

[CR54] Scholten S, Velten J, Bieda A (2017). Testing measurement invariance of the depression, anxiety, and stress scales (DASS-21) across four countries. Psychol Assess.

[CR55] Shakya HB, Christakis NA (2017). Association of Facebook use with compromised well-being: a longitudinal study. Am J Epidemiol.

[CR56] Sun Y, Zhang Y (2020). A review of theories and models applied in studies of social media addiction and implications for future research. Addict Behav.

[CR57] Swami V, Stieger S, Voracek M (2009). Psychometric evaluation of the Tagalog and German subjective happiness scales and a cross-cultural comparison. Soc Indic Res.

[CR58] Tromholt M (2016). The Facebook experiment: quitting Facebook leads to higher levels of well-being. Cyberpsychol Behav Soc Netw.

[CR59] Twenge JM (2019). More time on technology, less happiness? Associations between digital-media use and psychological well-being. Curr Dir Psychol Sci.

[CR60] Velten J, Lavallee KL, Scholten S (2014). Lifestyle choices and mental health: a representative population survey. BMC Psychol.

[CR61] Verduyn P, Lee DS, Park J (2015). Passive Facebook usage undermines affective well-being: experimental and longitudinal evidence. J Exp Psychol Gen.

[CR62] World Health Organization (2018) International classification of diseases for mortality and morbidity statistics (11th Revision). Available at: https://icd.who.int/browse11/l-m/en. Accessed 5 July 2022

[CR63] World Health Organization (2020) Physical Activity. Available at: https://www.who.int/news-room/fact-sheets/detail/physical-activity. Accessed 5 July 2022

[CR64] World Health Organization (2021) Coronavirus disease (COVID-19) pandemic. Available at: https://www.who.int/emergencies/diseases/novel-coronavirus-2019. Accessed 5 July 2022

[CR65] Zhao N, Zhou G (2021). COVID-19 stress and addictive social media use (SMU): mediating role of active use and social media flow. Front Psychiat.

[CR66] Zhong B, Huang Y, Liu Q (2021). Mental health toll from the coronavirus: social media usage reveals Wuhan residents’ depression and secondary trauma in the COVID-19 outbreak. Comput Hum Behav.

