# Supplementary material for: Experimental longitudinal evidence for causal role of social media use and physical activity in COVID-19 burden and mental health
Source: Z Gesundh Wiss. 2022 Sep 2:1–14. Online ahead of print. doi: 10.1007/s10389-022-01751-x (PMC9437404; doi:10.1007/s10389-022-01751-x)
Supplement: Supplementary file 1 — (DOCX 72 kb) [file 10389_2022_1751_MOESM1_ESM.docx]

Supplemental Material Table S1.

|  |  | Cronbach’s Alpha *α* | | | | | |
| --- | --- | --- | --- | --- | --- | --- | --- |
|  | Group | Baseline | Intermediate | Post | One-Month | Three-Month | Six-Month |
| Social Media Use Intensity | SM | .717 | .747 | .787 | .782 | .733 | .746 |
|  | PA | .810 | .793 | .803 | .803 | .798 | .725 |
|  | Combination | .786 | .819 | .830 | .814 | .839 | .829 |
|  | Control | .796 | .772 | .811 | .828 | .840 | .803 |
| Physical Activity Intensity | SM | .879 | .883 | .878 | .870 | .855 | .862 |
|  | PA | .831 | .802 | .826 | .862 | .838 | .854 |
|  | Combination | .827 | .805 | .809 | .828 | .837 | .828 |
|  | Control | .862 | .846 | .880 | .858 | .859 | .824 |
| Life Satisfaction | SM | .877 | .891 | .884 | .879 | .901 | .899 |
|  | PA | .879 | .907 | .921 | .896 | .900 | .911 |
|  | Combination | .886 | .896 | .875 | .909 | .906 | .874 |
|  | Control | .915 | .915 | .934 | .939 | .931 | .908 |
| Subjective Happiness | SM | .763 | .741 | .772 | .725 | .757 | .770 |
|  | PA | .743 | .747 | .781 | .813 | .748 | .809 |
|  | Combination | .758 | .788 | .704 | .780 | .722 | .700 |
|  | Control | .803 | .752 | .753 | .820 | .837 | .802 |
| Depression Symptoms | SM | .874 | .887 | .868 | .847 | .889 | .882 |
|  | PA | .859 | .864 | .872 | .892 | .877 | .900 |
|  | Combination | .889 | .854 | .849 | .837 | .838 | .789 |
|  | Control | .915 | .904 | .909 | .911 | .902 | .901 |
| Addictive Social Media Use | SM | .823 | .845 | .846 | .832 | .852 | .868 |
|  | PA | .844 | .866 | .871 | .888 | .875 | .872 |
|  | Combination | .847 | .858 | .859 | .865 | .866 | .894 |
|  | Control | .832 | .878 | .896 | .897 | .875 | .889 |
| COVID-19 Burden | SM | .832 | .827 | .807 | .853 | .859 | .759 |
|  | PA | .765 | .776 | .851 | .764 | .804 | .830 |
|  | Combination | .778 | .783 | .814 | .847 | .852 | .814 |
|  | Control | .773 | .819 | .823 | .892 | .875 | .804 |

*Reliability of the assessed instruments for each group and measurement time point (baseline to six months)*

*Notes*. Social Media (SM) Group: *N*=162, Physical Activity (PA) Group: *N*=161, Combination Group: *N*=159, Control Group: *N*=160; Baseline to Six-Months=measurement time points.
