# Supplementary material for: Experimental longitudinal evidence for causal role of social media use and physical activity in COVID-19 burden and mental health
Source: Z Gesundh Wiss. 2022 Sep 2:1–14. Online ahead of print. doi: 10.1007/s10389-022-01751-x (PMC9437404; doi:10.1007/s10389-022-01751-x)
Supplement: Supplementary file 2 — (DOCX 93 kb) [file 10389_2022_1751_MOESM2_ESM.docx]

Supplemental Material Table S2 (part 1).

*Pairwise comparisons of time points within groups (baseline to six months)*

|  |  | Baseline vs. Intermediate | | Baseline vs. Post | | Baseline vs. One-Month | | Baseline vs. Three-Month | | Baseline vs. Six-Month | | Intermediate vs. Post | | Intermediate vs. One-Month | | Intermediate vs. Three-Month | |
| --- | --- | --- | --- | --- | --- | --- | --- | --- | --- | --- | --- | --- | --- | --- | --- | --- | --- |
|  | Group | *p* | d_RM_ | *p* | d_RM_ | *p* | d_RM_ | *p* | d_RM_ | *p* | d_RM_ | *p* | d_RM_ | *p* | d_RM_ | *p* | d_RM_ |
| Daily Social Media Use Time (in Minutes) | SM | - | - | <.001 | .58 | <.001 | .40 | <.001 | .37 | <.001 | .41 |  |  |  |  |  |  |
|  | PA |  |  | .002 | .29 | <.001 | .31 | <.001 | .37 | <.001 | .49 |  |  |  |  |  |  |
|  | Combination |  |  | <.001 | .56 | <.001 | .63 | <.001 | .60 | <.001 | .66 |  |  |  |  |  |  |
|  | Control |  |  |  |  |  |  |  |  |  |  |  |  |  |  |  |  |
| Social Media Use Intensity | SM | <.001 | .37 | <.001 | .55 | <.001 | .52 | <.001 | .55 | <.001 | .47 | .005 | .30 |  |  | .039 | .26 |
|  | PA |  |  | .023 | .30 |  |  |  |  | .044 | .28 |  |  |  |  |  |  |
|  | Combination | <.001 | .46 | <.001 | .53 | <.001 | .51 | <.001 | .60 | <.001 | .55 |  |  |  |  | .019 | .25 |
|  | Control |  |  |  |  |  |  |  |  |  |  |  |  |  |  |  |  |
| Weekly Physical Activity Time (in Minutes) | SM |  |  |  |  |  |  | .021 | .37 |  |  |  |  |  |  |  |  |
|  | PA |  |  | <.001 | 1.17 | <.001 | .52 | <.001 | .52 |  |  |  |  |  |  |  |  |
|  | Combination |  |  | <.001 | 1.19 | <.001 | .74 | <.001 | .53 | <.001 | .66 |  |  |  |  |  |  |
|  | Control |  |  |  |  |  |  |  |  |  |  |  |  |  |  |  |  |
| Physical Activity Intensity | SM |  |  |  |  |  |  |  |  |  |  |  |  |  |  |  |  |
|  | PA | <.001 | .48 | <.001 | .43 | .002 | .31 | .007 | .29 | .028 | .26 |  |  |  |  |  |  |
|  | Combination | <.001 | .35 | <.001 | .38 | <.001 | .36 | <.001 | .32 | <.001 | .46 |  |  |  |  |  |  |
|  | Control |  |  |  |  |  |  |  |  |  |  |  |  |  |  |  |  |
| Life Satisfaction | SM |  |  | .003 | .30 | .013 | .26 | .012 | .28 |  |  |  |  |  |  |  |  |
|  | PA |  |  | .015 | .35 |  |  |  |  |  |  |  |  |  |  |  |  |
|  | Combination |  |  | <.001 | .39 | <.001 | .32 | .002 | .27 | <.001 | .29 | <.001 | .37 | .002 | .28 | .014 | .24 |
|  | Control |  |  |  |  |  |  |  |  |  |  |  |  |  |  |  |  |
| Subjective Happiness | SM |  |  |  |  | .019 | .26 |  |  |  |  |  |  | .022 | .24 |  |  |
|  | PA |  |  |  |  |  |  |  |  |  |  |  |  |  |  |  |  |
|  | Combination |  |  | .019 | .25 | .012 | .23 | <.001 | .38 | <.001 | .42 |  |  |  |  | .001 | .31 |
|  | Control |  |  |  |  |  |  |  |  |  |  |  |  |  |  |  |  |
| Depressive Symptoms | SM |  |  | .025 | .227 |  |  |  |  |  |  |  |  |  |  |  |  |
|  | PA |  |  | <.001 | .16 | .027 | .08 |  |  |  |  |  |  |  |  |  |  |
|  | Combination | <.001 | .41 | <.001 | .35 | <.001 | .35 | <.001 | .38 | <.001 | .36 |  |  |  |  |  |  |
|  | Control |  |  |  |  |  |  |  |  |  |  |  |  |  |  |  |  |
| Addictive Social Media Use | SM |  |  |  |  | .003 | .33 | .017 | .25 |  |  |  |  | .015 | .29 |  |  |
|  | PA |  |  |  |  |  |  |  |  |  |  |  |  |  |  | .001 | .39 |
|  | Combination | .005 | .25 | <.001 | .45 | <.001 | .41 | <.001 | .42 | <.001 | .31 | .003 | .26 | .006 | .23 | .005 | .24 |
|  | Control |  |  |  |  |  |  |  |  |  |  |  |  |  |  |  |  |
| COVID-19 Burden | SM |  |  |  |  | <.001 | .33 | <.001 | .35 | .003 | .27 |  |  | .009 | .30 | .003 | .33 |
|  | PA |  |  | .002 | .34 | <.001 | .47 | <.001 | .47 | <.001 | .47 |  |  | .001 | .35 | .001 | .36 |
|  | Combination |  |  |  |  | <.001 | .44 | <.001 | .38 | <.001 | .38 |  |  | <.001 | .43 | .006 | .34 |
|  | Control |  |  |  |  |  |  |  |  |  |  |  |  |  |  |  |  |
| Smoking Behavior (number of daily consumed tobacco products) | SM |  |  | .049 | .62 | .043 | .61 | .024 | .56 | .015 | .55 | <.001 | .91 | <.001 | .64 | .004 | .69 |
|  | PA |  |  |  |  |  |  |  |  |  |  |  |  |  |  |  |  |
|  | Combination |  |  |  |  |  |  |  |  |  |  |  |  |  |  |  |  |
|  | Control |  |  |  |  |  |  |  |  |  |  |  |  |  |  |  |  |

*Notes*. Social Media (SM) Group: *N*=162, Physical Activity (PA) Group: *N*=161, Combination Group: *N*=159, Control Group: *N*=160; exception: smoking behavior: SM Group: *n*=28, PA Group: *n*=24, Combination Group: *n*=25, Control Group: *n*=20; Baseline to Six-Month=measurement time points; *p*=significance; d_RM_=Cohen’s d_Repeated Measures_, effect-size measure of post-hoc comparisons within groups; pairwise comparisons are Bonferroni-corrected (*p*<.050, two-tailed).

|  |  | Intermediate vs. Six-Month | | Post vs. One-Month | | Post vs. Three-Month | | Post vs. Six-Month | | One-Month vs. Three-Month | | One-Month vs. Six-Month | | Three-Month vs. Six-Month | |
| --- | --- | --- | --- | --- | --- | --- | --- | --- | --- | --- | --- | --- | --- | --- | --- |
|  | Group | *p* | d_RM_ | *p* | d_RM_ | *p* | d_RM_ | *p* | d_RM_ | *p* | d_RM_ | *p* | d_RM_ | *p* | d_RM_ |
| Daily Social Media Use Time (in Minutes) | SM |  |  | .006 | .21 | <.001 | .22 |  |  |  |  |  |  |  |  |
|  | PA |  |  |  |  |  |  | .043 | .25 |  |  |  |  |  |  |
|  | Combination |  |  |  |  |  |  |  |  |  |  |  |  |  |  |
|  | Control |  |  |  |  |  |  |  |  |  |  |  |  |  |  |
| Social Media Use Intensity | SM |  |  |  |  |  |  |  |  |  |  |  |  |  |  |
|  | PA |  |  |  |  |  |  |  |  |  |  |  |  |  |  |
|  | Combination |  |  |  |  |  |  |  |  |  |  |  |  |  |  |
|  | Control |  |  |  |  |  |  |  |  |  |  |  |  |  |  |
| Weekly Physical Activity Time (in Minutes) | SM |  |  |  |  |  |  |  |  |  |  |  |  |  |  |
|  | PA |  |  | <.001 | .41 | <.001 | .37 | <.001 | .48 |  |  |  |  |  |  |
|  | Combination |  |  | <.001 | .40 | <.001 | .34 | .007 | .30 |  |  |  |  |  |  |
|  | Control |  |  |  |  |  |  |  |  |  |  |  |  |  |  |
| Physical Activity Intensity | SM |  |  |  |  |  |  |  |  |  |  |  |  |  |  |
|  | PA |  |  |  |  |  |  |  |  |  |  |  |  |  |  |
|  | Combination | .004 | .53 |  |  |  |  | .012 | .23 |  |  | .023 | .21 | .023 | .22 |
|  | Control |  |  |  |  |  |  |  |  |  |  |  |  |  |  |
| Life Satisfaction | SM |  |  |  |  |  |  |  |  |  |  |  |  |  |  |
|  | PA |  |  |  |  |  |  |  |  |  |  |  |  |  |  |
|  | Combination | .006 | .27 |  |  |  |  |  |  |  |  |  |  |  |  |
|  | Control |  |  |  |  |  |  |  |  |  |  |  |  |  |  |
| Subjective Happiness | SM |  |  |  |  |  |  |  |  |  |  |  |  |  |  |
|  | PA |  |  |  |  |  |  |  |  |  |  |  |  |  |  |
|  | Combination | <.001 | .32 |  |  |  |  | <.001 | .31 | .034 | .27 | <.001 | .31 |  |  |
|  | Control |  |  |  |  |  |  |  |  |  |  |  |  |  |  |
| Depressive Symptoms | SM |  |  |  |  |  |  |  |  |  |  |  |  |  |  |
|  | PA |  |  |  |  |  |  |  |  |  |  |  |  |  |  |
|  | Combination |  |  |  |  |  |  |  |  |  |  |  |  |  |  |
|  | Control |  |  |  |  |  |  |  |  |  |  |  |  |  |  |
| Addictive Social Media Use | SM |  |  |  |  |  |  |  |  |  |  |  |  |  |  |
|  | PA |  |  |  |  | .027 | .28 |  |  |  |  |  |  |  |  |
|  | Combination |  |  |  |  |  |  |  |  |  |  |  |  |  |  |
|  | Control |  |  |  |  |  |  |  |  |  |  |  |  |  |  |
| COVID-19 Burden | SM |  |  | .020 | .31 | .008 | .33 |  |  |  |  |  |  |  |  |
|  | PA | <.001 | .38 |  |  |  |  |  |  |  |  |  |  |  |  |
|  | Combination | .001 | .31 | .006 | .29 |  |  | .026 | .22 |  |  |  |  |  |  |
|  | Control |  |  |  |  |  |  |  |  |  |  |  |  |  |  |
| Smoking Behavior (number of daily consumed tobacco products) | SM | .012 | .64 |  |  |  |  |  |  |  |  |  |  |  |  |
|  | PA |  |  |  |  |  |  |  |  |  |  |  |  |  |  |
|  | Combination |  |  |  |  |  |  |  |  |  |  |  |  |  |  |
|  | Control |  |  |  |  |  |  |  |  |  |  |  |  |  |  |

Supplemental Material Table S2 (part 2).

*Pairwise comparisons of time points within groups (baseline to six months)*

*Notes*. Social Media (SM) Group: *N*=162, Physical Activity (PA) Group: *N*=161, Combination Group: *N*=159, Control Group: *N*=160; exception: smoking behavior: SM Group: *n*=28, PA Group: *n*=24, Combination Group: *n*=25, Control Group: *n*=20; Baseline to Six-Month=measurement time points; *p*=significance; d_RM_=Cohen’s d_Repeated Measures_, effect-size measure of post-hoc comparisons within groups; pairwise comparisons are Bonferroni-corrected (*p*<.050, two-tailed).
