# Supplementary material for: Experimental longitudinal evidence for causal role of social media use and physical activity in COVID-19 burden and mental health
Source: Z Gesundh Wiss. 2022 Sep 2:1–14. Online ahead of print. doi: 10.1007/s10389-022-01751-x (PMC9437404; doi:10.1007/s10389-022-01751-x)
Supplement: Supplementary file 3 — (DOCX 73 kb) [file 10389_2022_1751_MOESM3_ESM.docx]

Supplemental Material Table S3. *Pairwise comparisons between the four groups (baseline to six months)*

|  | Intermediate | | | Post | | | One-Month | | | Three-Month | | | Six-Month | | |
| --- | --- | --- | --- | --- | --- | --- | --- | --- | --- | --- | --- | --- | --- | --- | --- |
|  | Groups | *p* | d | Groups | *p* | d | Groups | *p* | d | Groups | *p* | d | Groups | *p* | d |
| Daily Social Media Use Time (in Minutes) |  |  |  | SM < Control | <.001 | .40 |  |  |  |  |  |  | SM < Control | .046 | .28 |
|  |  |  |  |  |  |  |  |  |  |  |  |  | PA < Control | .004 | .32 |
|  |  |  |  |  |  |  |  |  |  | Combination < SM | .011 | .39 |  |  |  |
|  |  |  |  | Combination < PA | .002 | .45 |  |  |  |  |  |  |  |  |  |
|  |  |  |  | Combination < Control | <.001 | .65 | Combination < Control | <.001 | .45 | Combination < Control | <.001 | .52 | Combination < Control | <.001 | .56 |
| Social Media Use Intensity | Combination < SM | .023 | .32 |  |  |  |  |  |  | Combination < SM | .036 | .32 | Combination < SM | .039 | .30 |
|  |  |  |  |  |  |  |  |  |  | Combination < PA | .042 | .30 | Combination < PA | .012 | .34 |
|  |  |  |  | Combination < Control | .030 | .31 | Combination < Control | <.001 | 42 | Combination < Control | <.001 | .49 | Combination < Control | <.001 | .48 |
| Weekly Physical Activity Time (in Minutes) |  |  |  | SM < PA | <.001 | .83 |  |  |  |  |  |  |  |  |  |
|  |  |  |  | SM < Combination | <.001 | .88 | SM < Combination | .005 | .38 |  |  |  | SM < Combination | .018 | .35 |
|  |  |  |  |  |  |  |  |  |  |  |  |  | PA < Combination | .040 | .31 |
|  |  |  |  | Control < PA | <.001 | .81 | Control < PA | .014 | .34 |  |  |  |  |  |  |
|  |  |  |  | Control < Combination | <.001 | .86 | Control < Combination | <.001 | .50 | Control < Combination | .035 | .32 | Control < Combination | .007 | .33 |
| Physical Activity Intensity | SM < PA | .020 | .32 | SM < PA | .028 | .32 |  |  |  |  |  |  |  |  |  |
|  |  |  |  | Control < PA | .019 | .33 |  |  |  |  |  |  |  |  |  |
|  | SM < Combination | .031 | .31 | SM < Combination | .031 | .31 |  |  |  |  |  |  | SM < Combination | <.001 | .46 |
|  |  |  |  |  |  |  |  |  |  |  |  |  | PA < Combination | .035 | .33 |
|  |  |  |  | Control < Combination | .021 | .33 | Control < Combination | .043 | .31 | Control < Combination | .010 | .35 | Control < Combination | <.001 | .53 |
| Life Satisfaction |  |  |  | Control < Combination | .006 | .37 | Control < Combination | .015 | .33 | Control < Combination | .046 | .30 | Control < Combination | .028 | .34 |
| Subjective Happiness |  |  |  |  |  |  |  |  |  | SM < Combination | .040 | .32 | SM < Combination | .012 | .38 |
|  |  |  |  |  |  |  |  |  |  | PA < Combination | .015 | .36 | PA < Combination | .002 | .43 |
|  |  |  |  |  |  |  |  |  |  | Control < Combination | .020 | .32 | Control < Combination | <.001 | .44 |
| Depression Symptoms | Combination < SM | .029 | .33 |  |  |  | Combination < SM | .043 | .33 | Combination < SM | .032 | .33 | Combination < SM | .006 | .40 |
|  | Combination < PA | .032 | .34 |  |  |  |  |  |  | Combination < PA | .048 | .33 |  |  |  |
|  | Combination < Control | .033 | .31 | Combination < Control | .036 | .31 | Combination < Control | .040 | .31 | Combination < Control | .013 | .35 | Combination < Control | .004 | .40 |
| Addictive Social Media Use |  |  |  | Combination < SM | .024 | .34 |  |  |  | Combination < SM | .038 | .31 | Combination < SM | .016 | .34 |
|  |  |  |  | Combination < PA | .032 | .32 |  |  |  |  |  |  |  |  |  |
|  |  |  |  | Combination < Control | .046 | .31 | Combination < Control | .042 | .31 | Combination < Control | .011 | .37 | Combination < Control | .014 | .35 |
| COVID-19 Burden |  |  |  |  |  |  | SM < Control | .002 | .40 | SM < Control | .001 | .41 | SM < Control | .005 | .39 |
|  |  |  |  |  |  |  | PA < Control | .031 | .32 | PA < Control | .035 | .32 | PA < Control | .010 | .35 |
|  |  |  |  |  |  |  | Combination < Control | <.001 | .50 | Combination < Control | <.001 | .46 | Combination < Control | <.001 | .53 |

*Notes*. Social Media (SM) Group: *N*=162, Physical Activity (PA) Group: *N*=161, Combination Group: *N*=159, Control Group: *N*=160; Baseline to Six-Month=measurement time points; *p*=significance; d=Cohen’s d, effect-size measure of post-hoc comparisons between groups; pairwise comparisons are Bonferroni-corrected (*p*<.050, two-tailed).
